# Remimazolam-flumazenil provides fast recovery from general anesthesia compared to propofol during radiofrequency catheter ablation of atrial fibrillation

**DOI:** 10.1038/s41598-024-63578-8

**Published:** 2024-06-03

**Authors:** Seohee Lee, Jaemoon Lee, So Yeong Hwang, Jae-Woo Ju, Karam Nam, Hyo-Jeong Ahn, So-Ryoung Lee, Eue-Keun Choi, Yunseok Jeon, Youn Joung Cho

**Affiliations:** 1 Department of Anesthesiology and Pain Medicine, Seoul National University Hospital, Seoul National University College of Medicine, 101 Daehak-ro, Jongno-gu, Seoul, 03080 Republic of Korea; 2 Department of Anesthesiology and Pain Medicine, Ajou University Hospital, Ajou University School of Medicine, Suwon, Republic of Korea; 3 Department of Anesthesiology and Pain Medicine, Konkuk University Hospital, Konkuk University College of Medicine, Seoul, Republic of Korea; 4 Department of Internal Medicine, Seoul National University Hospital, Seoul, Republic of Korea; 5 Department of Internal Medicine, Seoul National University College of Medicine, Seoul, Republic of Korea

**Keywords:** Drug safety, Pharmacology

## Abstract

The optimal anesthetic agent for radiofrequency catheter ablation (RFCA) of atrial fibrillation (AF) and its impact on the recovery profiles remain uncertain. We compared the recovery and hemodynamic parameters between the remimazolam-flumazenil and propofol groups during RFCA. Patients were randomized into the remimazolam-flumazenil and propofol groups. The primary outcome measure was the time to eye opening following the discontinuation of anesthetic agents. Secondary outcomes included time to extubation, time to discharge from the operating room, intraprocedural hemodynamic variables and postoperative quality outcomes. Fifty-three patients were included in the final analysis (n = 26 in the remimazolam-flumazenil and n = 27 in the propofol group). The time to eye opening was significantly shorter in the remimazolam-flumazenil group compared to the propofol group (median [interquartile range]: 174 [157–216] vs. 353 [230–483] s, *P* < 0.001). The mean blood pressure and bispectral index were significantly higher in the remimazolam-flumazenil group compared to the propofol group (mean difference [95% CI], 7.2 [1.7–12.7] mmHg and 6 [3–8]; *P* = 0.011 and < 0.001, respectively), which were within target ranges in both groups. Other secondary outcomes were comparable between the groups. Consequently, remimazolam emerges as a promising anesthetic agent, characterized by rapid recovery and stable hemodynamics, during RFCA of AF.

Trial registration: NCT05397886.

## Introduction

Radiofrequency catheter ablation (RFCA) is a well-established therapeutic modality for the management of symptomatic atrial fibrillation (AF). Considering the complex and time-intensive nature of cardiac ablative procedures, general anesthesia is the preferred approach for patients undergoing RFCA of AF using electroanatomical mapping^1,2^. Compared to sedation, general anesthesia offers improved patient immobility and catheter stability during prolonged and uncomfortable procedures, resulting in reduced arrhythmia recurrence rates and shorter fluoroscopy and procedure durations^3,4^. The increasing popularity of RFCA for AF under general anesthesia has led to an increased emphasis on rapid induction and early post-procedure recovery while maintaining hemodynamic stability, thereby optimizing the electrophysiology suite utilization and ensuring patient safety.

Propofol is commonly used as the primary anesthetic agent during cardiac catheterization and electrophysiology suites^5^. Although it induces excellent hypnosis and exerts minimal effects on cardiac electrophysiologic parameters^3^, it can lead to significant hypotension by suppressing cardiac function. Furthermore, propofol lacks antagonists, which can delay recovery from deep sedation or general anesthesia after prolonged procedures. Conversely, remimazolam, a novel ultrashort-acting intravenous benzodiazepine, is characterized by rapid onset, predictable maintenance, and availability of a reversal agent^6^; it demonstrates minimal hemodynamic instability^7^, even in elderly patients^8^. The effectiveness and feasibility of remimazolam have been established for endoscopic sedation and general anesthesia in several surgeries^9,10^. Moreover, remimazolam can be reversed with flumazenil, a benzodiazepine antagonist, leading to superior recovery profiles compared to propofol anesthesia^11,12^.

However, the recovery profiles and hemodynamic variables associated with remimazolam-flumazenil administration during general anesthesia for RFCA in patients with AF have not been well investigated. We hypothesized that remimazolam-flumazenil leads to faster recovery and superior hemodynamic stability than propofol for general anesthesia during RFCA of AF. Therefore, we compared the recovery profiles and hemodynamic variables between the patients who received remimazolam-flumazenil and those who received propofol for general anesthesia during RFCA of AF.

## Methods

### Ethical approval

This single center randomized-controlled trial was approved by the Institutional Review Board of Seoul National University Hospital (Seoul, Korea; approval no. 2203-070-1306; on 09/05/2022). The trial was registered at ClinicalTrials.gov on 31/05/2022 (NCT05397886; date of first registration, 02/08/2022). The study was conducted in accordance with the Declaration of Helsinki and the Good Clinical Practice guidelines. Informed consent was obtained from all participants; they were allowed to withdraw from the study at any point.

### Participants and randomization

We enrolled patients aged 20–75 years who were scheduled to undergo RFCA for AF under general anesthesia. Patients with a history of adverse or allergic reaction to the study drug, hypersensitivity to beans or peanuts, galactose intolerance, Lapp lactase deficiency, glucose-galactose malabsorption, severe hepatic or renal dysfunction, alcohol or drug dependency, organic brain disorder, spinal or cerebellar ataxia, acute narrow-angle glaucoma, shock, or comatose state were excluded. Furthermore, patients who were disoriented, hemodynamically unstable, pregnant or breastfeeding, refused to participate, or unable to remove the supraglottic airway or endotracheal tube at the end of the procedure and those who had used sedatives within 24 h were excluded.

We used a computer-generated randomization sequence and block randomization (blocks of four), conducted by an independent researcher to allocate patients to the remimazolam-flumazenil or propofol group in a 1:1 ratio. Independent nurses, who were not involved in the study, opened concealed envelopes, and prepared remimazolam-flumazenil or propofol based on the group allocation without labelling. Investigators (JML and SYH) blinded to the group assignment evaluated recovery profiles and postoperative outcomes. Furthermore, investigators (SL and YJC) blinded to group allocation analyzed the data. Anesthesia providers could not be blinded due to the differing appearances of the study drug formulations and the use of a reversal agent in the remimazolam-flumazenil group. However, enrolled patients were blinded to their group assignments, as the anesthetics were covered with a shield and the reversal agent was administered while the patients were anesthetized. Cardiac interventionists and data analysts remained blinded to the group allocation until the completion of the study.

### Study protocol

Patients were monitored using a three-lead electrocardiogram, pulse oxygen saturation (SpO_2_), non-invasive blood pressure (BP), and the bispectral index (BIS). Continuous monitoring of invasive arterial BP was performed when needed, based on the discretion of interventionists or anesthesiologists.

After preoxygenation with 100% oxygen, general anesthesia was induced using a continuous infusion of remimazolam or propofol, based on the group assignment. To ensure the prompt onset of the anesthetics and prevent any delays due to redundant infusion lines, we used three-way stopcock connectors, directly connected to the indwelling intravenous catheter, for administering each anesthetic agent. Remimazolam (Byfavo inj; Hana Pharmacy, Seoul, Korea) was administered initially at a rate for 6 mg/kg/h for induction of anesthesia, followed by a maintenance rate of 1–2 mg/kg/h. Conversely, propofol (FreSOfol MCT inj 2%; Fresenius Kabi Korea, Seoul, Korea) was administered by a target-controlled infusion (TCI), using a commercial infusion pump (Orchestra Base Primea, Fresenius-Vial, Sèvres, France) integrated with a March model, to achieve a target effect-site concentration (Ce) of 3–4 μg/mL. The infusion rates of the anesthetics were adjusted to maintain the BIS within the optimal range (40–60) for general anesthesia during the procedure. In both groups, remifentanil (Ultiva; GlaxoSmithKline, Brentford, UK) was administered by TCI, using a Minto model, to achieve a target Ce of 1–2 ng/mL; this approach provides effective analgesia and stable hemodynamics throughout the procedure.

Rocuronium (0.4–0.6 mg/kg) was administered to achieve neuromuscular blockade, then a supraglottic airway (i-gel™; Intersurgical LTD, Wokingham, Berkshire, UK) was inserted. Proper placement of the supraglottic airway was confirmed by observing bilateral chest rise during ventilation, adequate capnography, and the absence of an audible leak at an airway pressure ≥ 10 cmH_2_O. In cases where the proper placement of the supraglottic airway was not achieved, endotracheal intubation was performed. Mechanical ventilation was provided with a tidal volume of 0.6–0.8 mL/kg, a ventilation rate of 10–12/min, a fraction of inspired oxygen of 0.4–0.5, and a positive end-expiratory pressure of 4–5 cmH_2_O. Moreover, esophageal temperature monitoring was performed.

Intraprocedural hypotension was defined as three consecutive readings of systolic BP < 80 mmHg detected at a 2.5-min interval between the initiation and completion of the ablative procedure. During ablation, intraprocedural hypotension was managed with bolus or continuous administration of vasoactive agents, at the discretion of anesthesiologists. In most cases, continuous norepinephrine infusion was preferred, except for patients with preoperative left ventricular dysfunction. The remaining perioperative management was carried out in accordance with the standard anesthetic protocols established at our institution.

Upon completion of the ablative procedure, the administration of intravenous anesthetics was discontinued, and the percutaneously accessed vessels were closed. To reverse neuromuscular blockade, intravenous sugammadex was administered at a dose of 2–4 mg/kg following the discontinuation of the anesthetic agents. In the remimazolam group, flumazenil (0.2 mg) was administered immediately after the administration of sugammadex, to antagonize the effects of remimazolam. If adequate arousal was not observed, additional doses of flumazenil (0.1 mg) were administered repeatedly at 1-min intervals, up to a maximum total dose of 1.0 mg, after 2 min of the initial administration of flumazenil.

The time to eye opening was measured as the duration between the cessation of infusion of each anesthetic and the initial eye opening in response to a verbal command of “open your eyes” and upon calling the patient’s name with a light touch. Similarly, the time to extubation was recorded as the duration between the cessation of each anesthetic and the removal of the supraglottic airway or endotracheal tube, ensuring that the patient had adequate spontaneous respiration. The times to eye opening and extubation were assessed by investigators (JL and SL) blinded to the group assignment. Following arousal from anesthesia, patients were monitored in the cardiovascular care unit; subsequently, they were transferred to general wards unless any immediate post-procedural complications were observed.

### Radiofrequency catheter ablation of atrial fibrillation

The RFCA was performed using irrigation catheters (Thermocool SmartTouch SF D-F, Biosense Webster Inc.; QDOT Micro, Biosense Webster Inc.). All procedures including this study used the CARTO system (Biosense Webster Inc.), which used integrated respiratory gating and gathered 3-dimensional anatomical data. Circumferential pulmonary vein (PV) isolation was performed with systemic anticoagulation achieved through intravenous heparin administration, maintaining an activated clotting time of 350–400 s. Ablation other than PV antrum was performed at the discretion of the operator. After PV isolation, intravenous infusion of isoproterenol was administered to induce AF and evaluate the presence of non-PV trigger. Patients who remained in AF after the procedure underwent electrical cardioversion.

### Study outcomes and data collection

The primary outcome measure was the time to eye opening following the discontinuation of anesthetic agents. Secondary outcomes included the time to extubation, time between the administration of anesthetic agents and loss of consciousness, intraprocedural hemodynamic variables and BIS, incidence of intraprocedural hypotension, use and maximum dose of vasopressors administered to manage intraprocedural hypotension, assessment using the Korean version of 15-item Quality of Recovery score^13^ at 24 h, the length of hospital stay, and postoperative quality outcomes, such as nausea, vomiting, and delirium. Postoperative outcome variables were assessed by investigators (J-WJ and KN) blinded to the group assignment.

Baseline characteristics, comorbidities, current medications and preoperative laboratory findings of the patients were collected. Intraprocedural hemodynamic variables and BIS were recorded at baseline, after anesthesia induction, at 60 min post-induction, upon procedure completion, and after anesthesia arousal. Following the initial pass of PV antral ablation, we evaluated the residual potential in predefined PV antral segments. If any residual potential was detected, we performed additional supplementary ablation. After a 20-min observation period to confirm PV isolation achievement, we assessed for early PV reconnection, defined as a composite of residual potential and early PV reconnection.

### Sample size calculation

To calculate the sample size, we referred to a previous study where the mean ± standard deviation of time to eye opening after discontinuation of propofol infusion was 10.3 ± 5.1 min^10^. A clinically significant reduction in the time to eye opening was defined as a change of 40%. Based on the power of 80% and an alpha error of 5%, the power analysis revealed that 27 patients were required in each group for comparison using an independent *t*-test. Considering a 5% dropout rate, we enrolled 54 patients in this study.

### Statistical analysis

Data are presented as means ± standard deviations, medians (interquartile ranges), numbers (percentages), or mean differences (95% confidence interval [CI]). Continuous variables were analyzed using Student’s *t*-test or Mann–Whitney *U* test, whereas categorical variables were assessed using the chi-squared test or Fisher’s exact test. The normality of data was assessed using the Shapiro–Wilk test. Repeated measures were analyzed using a linear mixed-effects model, which incorporated independent fixed effects of the group, measurement time, and their interaction. Additionally, a random effect of the patient (a random intercept) was included using a compound symmetry covariance structure. In cases where the interaction between the group and measurement time was significant, we compared the mean difference between the groups at each time point, such as post-anesthesia induction, 60 min post-induction, upon procedure completion, and after arousal from anesthesia, using linear contrast in the linear mixed-effects model. Furthermore, the *P* value from the linear contrast test was adjusted using Bonferroni correction (multiplied by 4), to account for multiple comparisons. Statistical analyses were performed using SPSS (ver. 25.0; IBM Corp., Armonk, NY, USA) and R software (ver.4.1.0; R Development Core Team, Vienna, Austria). *P* values < 0.05 were considered statistically significant.

## Results

**Figure 1:**
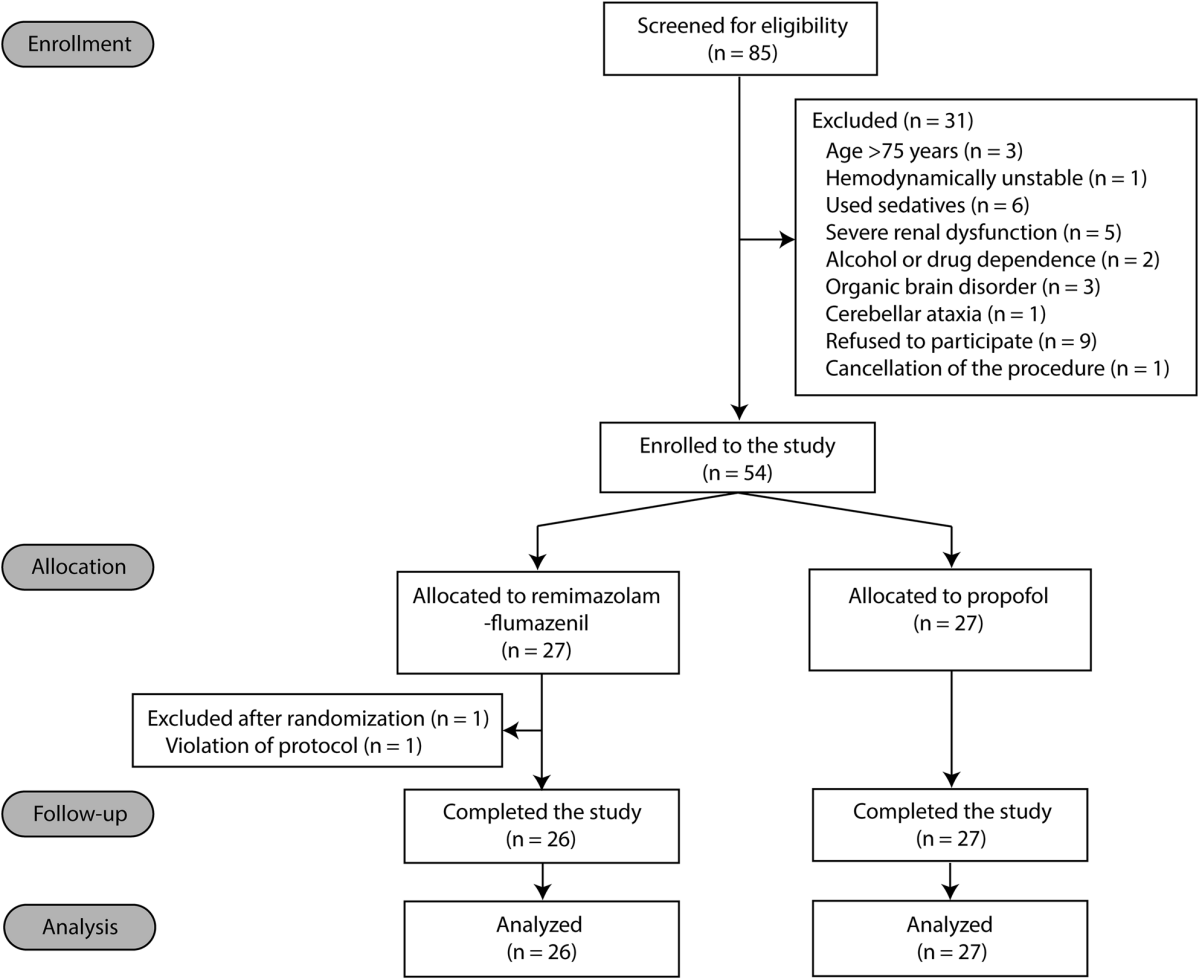
CONSORT diagram.

Of the 85 patients who underwent screening, 31 were excluded and 54 were randomized into the remimazolam-flumazenil (n = 27) and propofol (n = 27) groups between 2 August, 2022, and 25 April, 2023 (Fig. 1). Notably, one patient from the remimazolam-flumazenil group was excluded post-enrolment due to non-compliance with the study protocol, particularly a failure to administer flumazenil in a timely manner after discontinuation of remimazolam. Consequently, the final analysis enrolled 53 patients, with 26 in the remimazolam-flumazenil group and 27 in the propofol group. Baseline characteristics of patients and intraprocedural variables were comparable between the groups (Tables 1, 2).

**Table 1:** Baseline characteristics of patients undergoing radiofrequency catheter ablation for atrial fibrillation under general anesthesia using remimazolam-flumazenil or propofol.

	Remimazolam-flumazenil group (n = 26)	Propofol group (n = 27)
*Baseline characteristics*		
Age (years)	66 (61–69)	58 (48–66)
Male	20 (77%)	22 (82%)
Height (cm)	167.8 (8.5)	168.6 (9.0)
Weight (kg)	69.9 (14.4)	72.6 (14.9)
Body mass index (kg/m^2^)	24.6 (3.8)	26.1 (2.8)
Smoking history		
Never	12 (46%)	16 (59%)
Current	8 (31%)	6 (22%)
Ex-smoker	6 (23%)	5 (19%)
*Comorbidities*		
Hypertension	13 (50%)	9 (33%)
Diabetes mellitus	6 (23%)	1 (4%)
Hyperlipidemia	9 (35%)	4 (15%)
Coronary artery disease	1 (4%)	1 (4%)
Myocardial infarction	1 (4%)	0 (0%)
Congestive heart failure	1 (4%)	0 (0%)
Pulmonary hypertension	0 (0%)	0 (0%)
Chronic obstructive lung disease	0 (0%)	0 (0%)
Peripheral artery disease	0 (0%)	0 (0%)
*Current medication*		
Aspirin	1 (4%)	1 (4%)
Warfarin	1 (4%)	2 (7%)
NOAC	25 (96%)	25 (93%)
Apixaban	12 (46.2%)	7 (26%)
Dabigatran	1 (4%)	3 (11%)
Edoxaban	3 (12%)	4 (15%)
Rivaroxaban	9 (35%)	11 (41%)
ß blocker	17 (65%)	12 (44%)
Calcium channel blocker	6 (23%)	8 (30%)
Diuretics	6 (23%)	4 (15%)
Statin	7 (27%)	9 (33%)
Oral hypoglycemic agents	6 (23%)	2 (7%)
Insulin	0 (0%)	0 (0%)
Nitrate	1 (9%)	0 (0%)
Digoxin	2 (8%)	2 (7%)
Anti-arrhythmic agents	23 (89%)	21 (78%)
Amiodarone	4 (15%)	7 (26%)
Dronedarone	1 (4%)	1 (4%)
Flecainide	8 (31%)	4 (15%)
Pilsicainide	2 (8%)	0 (0%)
Propafenone	8 (31%)	9 (33%)
*Preoperative laboratory findings*		
Hematocrit (%)	43 (5)	43 (4)
Creatinine (mg/dL)	0.90 (0.76–0.97)	0.85 (0.78–1.01)
LVEF (%)	57 (56–63)	58 (56–62)
LA volume (mL)	87 (27)	85 (25)

Data are presented as median (interquartile range), number (%) or value (standard deviation).

ASA PS, American Society of Anesthesiologists Physical Status; NOAC, Non-Vitamin K antagonist oral anticoagulants; LVEF, left ventricular ejection fraction; LA, left atrium.

**Table 2:** Intraoperative variables in patients received remimazolam-flumazenil or propofol during radiofrequency catheter ablation of atrial fibrillation.

	Remimazolam-flumazenil group (n = 26)	Propofol group (n = 27)	*P*
Time to loss of consciousness (s)	101 (84–114)	121 (70–161)	0.328
Time to eye opening (s)	174 (157–216)	353 (230–483)	< 0.001
Time to extubation (s)	211 (177–268)	382 (308–572)	< 0.001
Duration of procedure (min)	142 (39)	135 (42)	0.532
Total infused remifentanil (μg)	656 (271)	623 (170)	0.604
Total administered propofol (mg)		1195 (268)	NA
Total administered remimazolam (mg)	354 (108)		NA
Total administered flumazenil (mg)	0.3 (0.3–0.4)		NA
Total infused crystalloid (mL)	600 (475–763)	600 (350–700)	0.447
Urine output (mL)	400 (233–915)	650 (300–855)	0.552
Incidence of intraprocedural hypotension*	3 (12%)	1 (4%)	0.341
Use of norepinephrine during procedure	22 (85%)	24 (89%)	0.704
Maximum dosage of norepinephrine administration (μg/kg/min)	0.04 (0.02–0.06)	0.04 (0.02–0.07)	0.641
Duration of norepinephrine infusion (min)	94 (66)	107 (64)	0.458

Data are presented as median (interquartile range), mean (SD), or number (%). NA, not applicable.

*Intraoperative hypotension was defined when three consecutive systolic blood pressure < 80 mmHg at 2.5 min-interval was detected.

**Figure 2:**
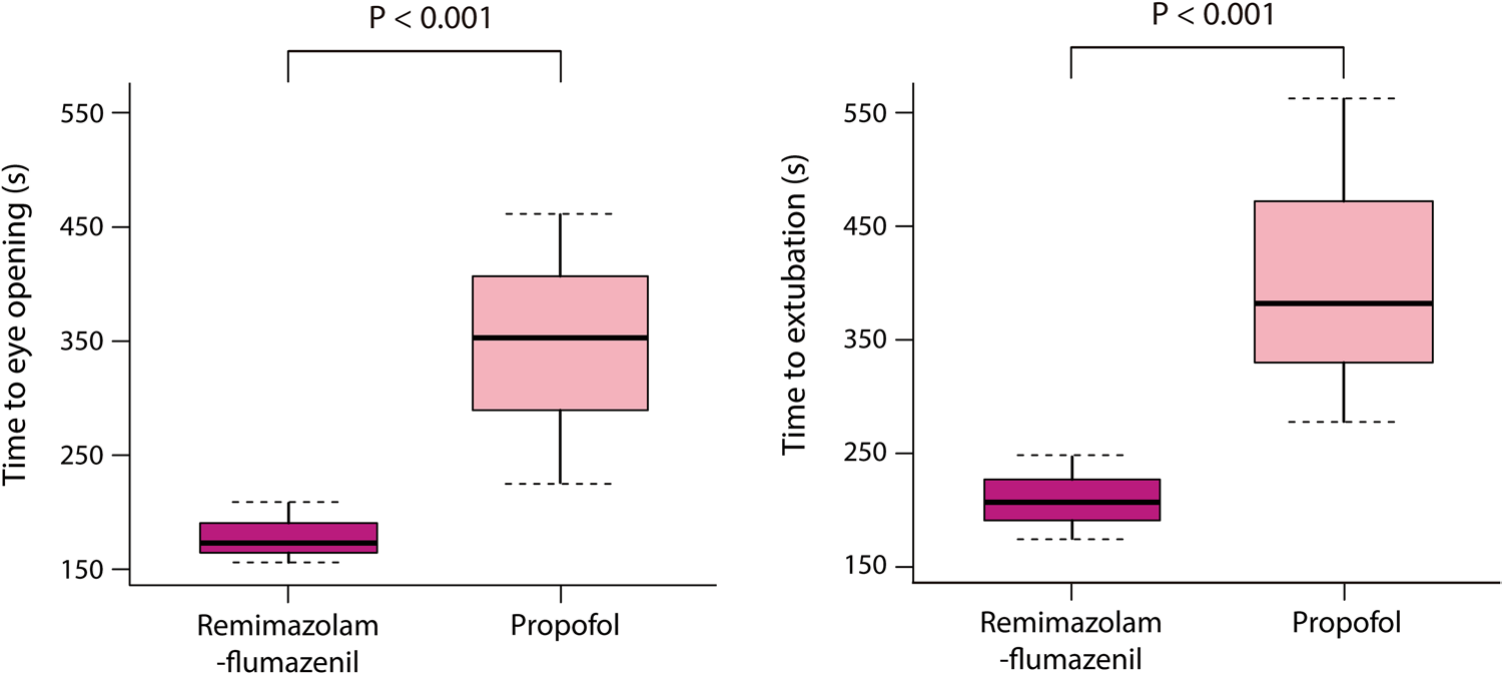
Boxplots of time to eye opening and extubation in patients received remimazolam-flumazenil or propofol during radiofrequency catheter ablation of atrial fibrillation. Horizontal line within the box indicates the median value; lower and upper boundaries of the box indicate the 25th and 75th percentiles, respectively; horizontal dotted lines outside the box indicate the minimum and maximum values of the data.

The median time to loss of consciousness was similar between the groups (Table 2). However, the time to eye opening and time to extubation were significantly shorter in the remimazolam-flumazenil group compared to the propofol group (median [interquartile range]: 174 [157–216] vs. 353 [230–483] s and 211 [177–268] vs. 382 [308–572] s, respectively, both *P* < 0.001; Fig. 2).

**Figure 3:**
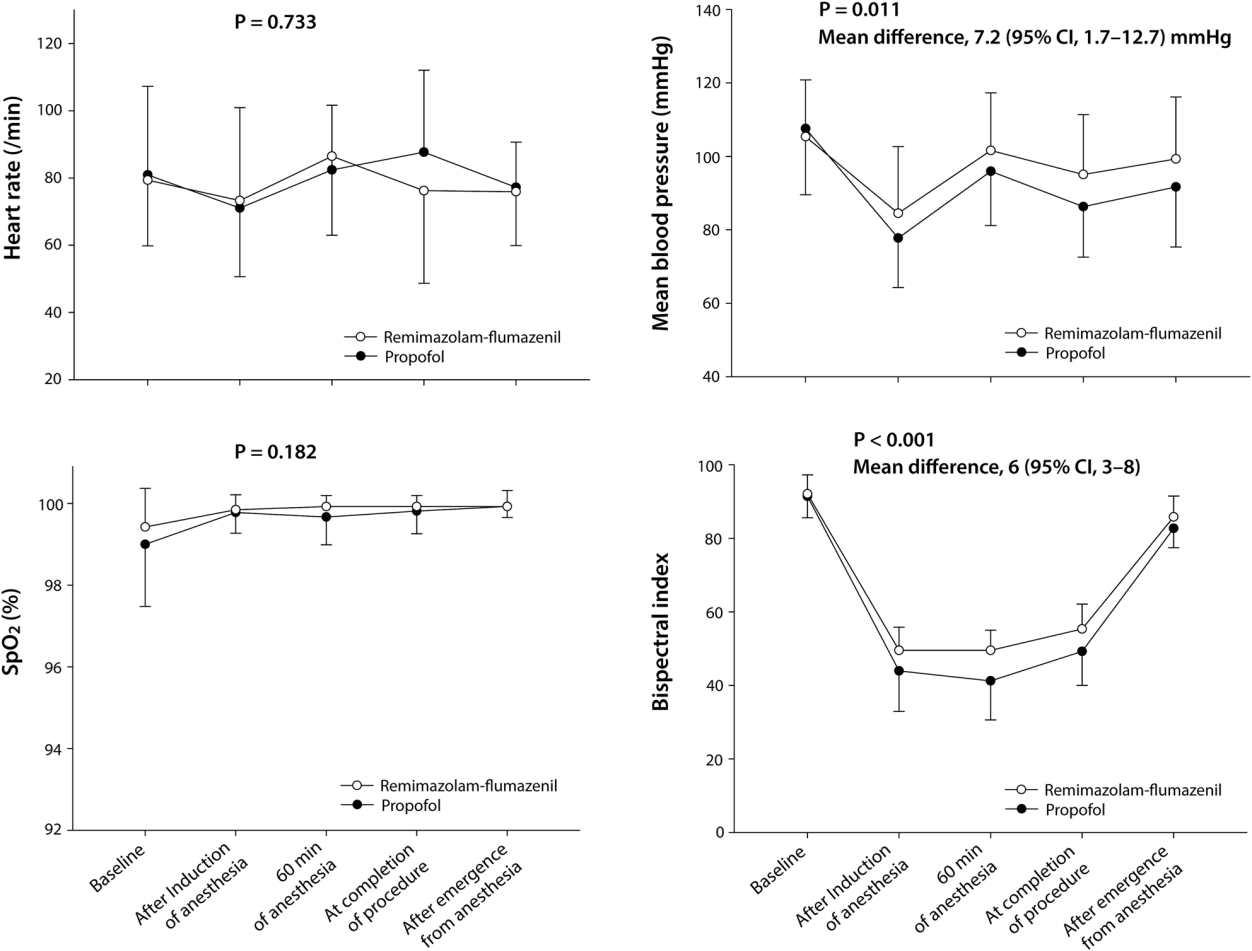
Hemodynamic variables and bispectral index in patients received remimazolam-flumazenil or propofol during radiofrequency catheter ablation of atrial fibrillation. Data points and error bars represent means and standard deviations, respectively. SpO_2_, pulse oxygen saturation. There were eight missing values for bispectral index (each two in the remimazolam-flumazenil and propofol groups at baseline and after emergence from anesthesia, respectively).

No significant differences were observed in baseline heart rate, mean BP, SpO_2_, and BIS between the groups (*P* = 0.715, 0.635, 0.485, and 0.920, respectively). Similarly, no significant differences were observed between the groups in terms of heart rate and SpO_2_ during the procedure (*P* = 0.733 and 0.182, respectively). However, mean BP and BIS were significantly higher in the remimazolam-flumazenil group compared to the propofol group (mean difference [95% CI], 7.2 [1.7–12.7] mmHg, *P* = 0.011 and 6 [3–8], *P* < 0.001, respectively; Fig. 3). No significant interactions were observed between the groups for measurement time for heart rate, mean BP, SpO_2_ and BIS (*P* = 0.097, 0.952, 0.360, and 0.240, respectively).

The incidence and duration of hypotension and the use and maximum dose of norepinephrine infusion exhibited no significant differences between the groups (Table 2). Additionally, no complications were reported related to the administration of study drugs; furthermore, no instances of awareness or recall were reported.

Table 3 presents the postoperative quality outcomes. No significant differences were observed between the groups in terms of postoperative nausea, vomiting, and delirium, 15-item Quality of Recovery scores, and the length of hospital stay. The rates of acute PV reconnection were comparable between the two groups without statistically significant difference (*P* = 0.069).

**Table 3:** Postoperative quality outcomes in patients received remimazolam-flumazenil or propofol during radiofrequency catheter ablation of atrial fibrillation.

	Remimazolam-flumazenil group (n = 26)	Propofol group (n = 27)	*P*
Postoperative nausea and vomiting	1 (4%)	3 (11%)	> 0.999
Postoperative delirium	0 (0%)	0 (0%)	NA
QoR-15 K score*	137 (127–145)	144 (139–145)	0.130
Length of hospital stay (d)	2 (2–2)	2 (2–2)	0.171
Acute PV reconnection^†^	11 (48%)	6 (23%)	0.069

Data are presented as number (%) or median (interquartile range). NA, not applicable, QoR-15 K, Korean version of 15-item Quality of Recovery questionnaire; PV pulmonary vein.

*One missing value in the propofol group.

^†^Three cases in the remimazolam-flumazenil group and a case in the propofol group were not evaluated for pulmonary vein reconnection during the procedure.

## Discussion

We observed that patients who received remimazolam-flumazenil demonstrated a significantly shorter time to eye opening and extubation following the discontinuation of anesthetic agents compared to those who received propofol during RFCA of AF, while no differences were observed in postoperative quality outcomes. Furthermore, the remimazolam-flumazenil group exhibited a statistically significant elevation in mean arterial pressure compared to the propofol group. BIS was maintained within the target range during general anesthesia in both groups; however, it was higher in the remimazolam-flumazenil group compared to the propofol group.

Although remimazolam has demonstrated favorable anesthetic and recovery profiles for short procedures^14^, most studies evaluating its effectiveness and safety have focused on endoscopies^15,16^ or surgeries in patients without cardiac diseases^10^. Therefore, the generalizability of these findings to anesthesia during AF ablation remains limited. We conducted a randomized trial that compared the effects of remimazolam-flumazenil and propofol in patients with AF who underwent RFCA; this procedure is associated with the potential for hemodynamic instability, prolonged procedural durations and necessitates patient immobility.

Propofol is the most commonly used sedative and hypnotic agent for procedural anesthesia^17^. Although propofol is widely used for moderate to deep sedation and general anesthesia, its narrow therapeutic window can lead to hypotension and respiratory depression at Ce close to that used for ambulatory sedation^18^. Conversely, propofol can decelerate fibrillation activity by modulating ionic currents or parasympathetic tone during profound sedation in AF^19^. However, the definitive impact of propofol on ablation outcomes remains uncertain. Although we did not assess the effects of remimazolam on the modulation of the autonomic nervous system or arrhythmia, remimazolam can be a promising alternative to propofol for anesthesia during RFCA of AF.

Remimazolam has demonstrated more stable hemodynamics than propofol and other anesthetic agent^8,11,15,16,20^. Consistent with previous studies, we observed significantly higher mean BP during AF ablation in the remimazolam-flumazenil group compared to the propofol group, although there were no differences in the use of norepinephrine or the maximum dosage of norepinephrine administered during the procedure (Fig. 3). Additionally, a recent meta-analysis demonstrated that remimazolam provides more stable hemodynamic parameters than propofol during endoscopic sedation^21^. Notably, remimazolam has been used for general anesthesia during high-risk cardiovascular interventions, such as MitraClip^®^ implantation in a patient with severe mitral regurgitation and transcatheter aortic valve replacement for severe aortic stenosis^22,23^. In these cases, the remimazolam group demonstrated effective anesthesia management for patient with severe mitral regurgitation and advanced heart failure with a very low ejection fraction^22^, and reduced need for a vasopressor, compared to propofol/sevoflurane or midazolam/propofol groups^23^. Therefore, remimazolam, characterized by rapid onset and offset, predictable maintenance and minimal hemodynamic instability, is a promising choice for procedural sedation or general anesthesia in patients undergoing high-risk cardiovascular intervention.

In our study, the remimazolam-flumazenil group demonstrated significantly higher BIS values than the propofol group, consistent with previous studies^10^. Although BIS monitoring during remimazolam administration exhibited greater variability with relatively higher values, simultaneous assessments of neurological sedative indicators, including spectral edge frequency (SEF) and resting pupil diameter, confirmed an adequate sedation level during remimazolam anesthesia^24^. The unprocessed electroencephalogram, SEF, is generally known as a more accurate sedative indicator. Anesthetized patients exhibited a wider range of SEF values during induction and maintenance with remimazolam^24^, primarily due to increased β waves^25^, which is considered to attribute to higher BIS values during remimazolam anesthesia compared to propofol. The resting pupil diameter, a traditional indicator of the depth of anesthesia, was under 2 mm during remimazolam anesthesia according to a previous report^24^. This measurement is comparable to diameters observed during anesthesia with sevoflurane, desflurane, or propofol^26^, despite some patients having a BIS > 60 intraoperatively with remimazolam. Therefore, we cannot directly compare the depth of anesthesia using BIS values between anesthesia with remimazolam and propofol, and any differences in BIS may have minimal influence on the primary outcome of this study. We observed no instances of awakening, awareness, or recall in either group. However, a sustained elevation in BIS, even with the administration of appropriate dosages of anesthetic agents, can lead anesthesiologists to consider the overdose of these agents; therefore, a comprehensive understanding of the distinctive features of BIS monitoring is essential.

In this study, the choice of vasopressor during electrophysiological studies was based on previous literature and institutional preference. Although phenylephrine is a selective α_1_-agonist, its infusion increased atrial refractoriness and caused intra-atrial conduction delays in patients undergoing electrophysiological studies^27^. Phenylephrine can effectively terminate supraventricular tachycardia and is associated with sinus bradycardia^28^. It also exhibits differential effects in atrial and ventricular tissues^28^, and the induction of AF during ablative therapy is influenced by non-uniform changes in atrial refractoriness during phenylephrine infusion^27^. Norepinephrine, which has both α- and β-agonistic effects, increases the sinus cycle length and prolongs atrial and ventricular refractoriness^29^. Depression of sinus and atrioventricular nodal function by norepinephrine is considered to result from the direct effects of α-adrenergic stimulation or the indirect effects of increased vagal tone, attributable to increased BP and activation of baroreceptors^29^. Both norepinephrine and phenylephrine prolonged the action potential of normal sheep Purkinje fibers, with the effect of phenylephrine being greater than that of norepinephrine^30^. Moreover, the prolongation of the functional refractory period was greater with phenylephrine administration than with norepinephrine in the isolated heart^31^. Nevertheless, both intravenous vasopressors appear relatively safe with respect to proarrhythmic potential in humans^28^.

This study had several limitations. First, although the rate of acute PV reconnection was not a predetermined outcome of this study and did not reach statistical significance, given the potential influence of various anesthetics on arrhythmia and neural activity, further comprehensive evaluation of this topic may be essential in the future studies. Second, significant individual pharmacokinetic variability and the re-emergence of remimazolam effects following flumazenil reversal have been previously documented^32,33^. Moreover, although agitation is a well-known complication of flumazenil administration, no such complications were observed following remimazolam anesthesia during electrophysiological studies in our study cohort, similar to previous reports^34,35^. Despite these findings, our study confirmed the benefits of flumazenil use in expediting the regaining of consciousness without any instances of re-sedation or agitation. Nevertheless, anesthesiologists should remain vigilant about the potential for re-sedation or agitation following remimazolam-flumazenil anesthesia. Third, although we included patients with symptomatic arrhythmia, the preoperative left ventricular performance and hemodynamic parameters of the patients were within normal range. Considering the preoperative status of the patients in our study, the beneficial effects of remimazolam may not have been fully elucidated, particularly in patients with hemodynamic instability. Further validation studies in hemodynamically unstable patients, such as those with sustained ventricular arrhythmia, could provide further insights into the distinctive characteristics of remimazolam. Fourth, flumazenil was administered to reverse the sedative effects of benzodiazepine (remimazolam) according to the current study protocol. Although flumazenil was administered to every patient in the remimazolam group, we cannot exclude the possibility that its use influenced the observed difference in recovery time. However, the administration of flumazenil was considered due to the quick turnover time of procedures and safe emergence from anesthesia, as supported by previous studies^11,12^. The current study demonstrated that remimazolam, in combination with sequential flumazenil, may be a preferable alternative to propofol alone in patients undergoing RFCA for AF, with respect to speed of recovery and stable hemodynamics. Nevertheless, we do not recommend the routine use of an antagonist to facilitate recovery from anesthesia; its administration should be based on individual clinical needs. Lastly, although there were numerically higher incidences of hypotension (3/26 [12%] vs. 1/27 [4%]) and PV reconnection rates (11/23 [48%] vs. 6/26 [23%]) in the remimazolam-flumazenil group compared to the propofol group, these differences were not statistically significant (Tables 2, 3). However, the size of this study is insufficient to conclusively assess the important outcome of post-procedural adverse events. Therefore, we cannot completely rule out the potential risk of sustained hypotension or PV reconnection rate in some patients anesthetized with remimazolam, and further large-scale, multicenter studies are necessary.

In conclusion, the remimazolam-flumazenil group demonstrated a significantly shorter time to eye opening and extubation following the discontinuation of anesthetic agents compared to the propofol group in patients undergoing RFCA of AF under general anesthesia. Consequently, remimazolam emerges as a promising anesthetic agent, characterized by rapid recovery and stable hemodynamics, during RFCA of AF.

## Data Availability

The datasets generated and/or analyzed during this study are available from the corresponding author on reasonable request.
